# *Streptomyces griseus* KJ623766: A Natural Producer of Two Anthracycline Cytotoxic Metabolites β- and γ-Rhodomycinone

**DOI:** 10.3390/molecules26134009

**Published:** 2021-06-30

**Authors:** Ahmed S. Abu Zaid, Ahmed E. Aleissawy, Ibrahim S. Yahia, Mahmoud A. Yassien, Nadia A. Hassouna, Khaled M. Aboshanab

**Affiliations:** 1 Department of Microbiology and Immunology, Faculty of Pharmacy, Ain Shams University, Organization of African Unity St, Abbassia, Cairo P.O. Box 11566, Egypt; ahmed.abouzid@pharma.asu.edu.eg (A.S.A.Z.); mahmoud.yassien@pharma.asu.edu.eg (M.A.Y.); nadia.hassouna@pharma.asu.edu.eg (N.A.H.); 2 Department of Pharmacognosy, Faculty of Pharmacy, Ain Shams University, Organization of African Unity St, Abbassia, Cairo P.O. Box 11566, Egypt; aelissawy@pharma.asu.edu.eg; 3 Research Center for Advanced Materials Science (RCAMS), King Khalid University, Abha P.O. Box 9004, Saudi Arabia; ihussein@kku.edu.sa; 4 Advanced Functional Materials & Optoelectronic Laboratory (AFMOL), Department of Physics, Faculty of Science, King Khalid University, Abha P.O. Box 9004, Saudi Arabia; 5 Nanoscience Laboratory for Environmental and Bio-Medical Applications (NLEBA), Semiconductor Lab., Physics Department, Faculty of Education, Ain Shams University, Cairo P.O. Box 11757, Egypt

**Keywords:** cytotoxic activity, fermentation, *Streptomyces griseus*, β-rhodomycinone, γ-rhodomycinone

## Abstract

Background: This study aimed to produce, purify, structurally elucidate, and explore the biological activities of metabolites produced by *Streptomyces (S.) griseus* isolate KJ623766, a recovered soil bacterium previously screened in our lab that showed promising cytotoxic activities against various cancer cell lines. Methods: Production of cytotoxic metabolites from *S. griseus* isolate KJ623766 was carried out in a 14L laboratory fermenter under specified optimum conditions. Using a 3-(4,5-dimethylthazol-2-yl)-2,5-diphenyl tetrazolium-bromide assay, the cytotoxic activity of the ethyl acetate extract against *Caco*2 and *Hela* cancer cell lines was determined. Bioassay-guided fractionation of the ethyl acetate extract using different chromatographic techniques was used for cytotoxic metabolite purification. Chemical structures of the purified metabolites were identified using mass, 1D, and 2D NMR spectroscopic analysis. Results: Bioassay-guided fractionation of the ethyl acetate extract led to the purification of two cytotoxic metabolites, R1 and R2, of reproducible amounts of 5 and 1.5 mg/L, respectively. The structures of R1 and R2 metabolites were identified as β- and γ-rhodomycinone with CD_50_ of 6.3, 9.45, 64.8 and 9.11, 9.35, 67.3 µg/mL against *Caco*2, *Hela* and *Vero* cell lines, respectively. Values were comparable to those of the positive control doxorubicin. Conclusions: This is the first report about the production of β- and γ-rhodomycinone, two important scaffolds for synthesis of anticancer drugs, from *S. griseus*.

## 1. Introduction

Worldwide cancer incidence and mortality have always been a concern to the community. The cancer mortality rate has generally declined over the years; however, there is still an increased mortality rate in poorer countries that receives considerable attention from healthcare professionals [1]. This suggests the importance of prompt detection, effective treatment, and successful prevention strategies [1]. Natural products, particularly microbial metabolites, have been the mainstay of cancer chemotherapy. These products could provide many of the lead structures that can be used for production of new derivatives with improved biological activities [2]. These chemotherapeutic agents isolated from plant, marine, and bacterial sources are commonly used in chemotherapy as well as to treat lupus and juvenile rheumatoid arthritis [3].

Microbial metabolites can be utilized as novel anticancer agents; with fewer side effects [4]. Streptomycetes have been the center of attraction within the scientific community owing to their capability to produce various bioactive compounds, for instance, with different antimicrobial, anticancer, and antioxidant properties [5].

Various members of *Streptomyces* have made imperative contributions to human health with their capabilities to produce various important active metabolites, including anticancer agents [6]. Many drugs such as bleomycins, dactinomycin, mitomycin C, daunomycin, and doxorubicin originating from Streptomycetes are currently used in the treatment of cancer, and most of them were introduced into the clinic before discovering their modes of actions [2]. Doxorubicin is an antibiotic derived from the *Streptomyces peucetius* bacterium. It has widespread use as a chemotherapeutic agent since the 1960s [7], Unfortunately, Doxorubicin also can cause cardiotoxicity, with patients only allowed a cumulative lifetime dose of 550 mg/m^2^ [8]. Doxorubicin is part of the anthracycline group of chemotherapeutic agents [7].

Anthracyclines are drugs extracted from *Streptomyces spp*. and used in the treatment of various types of cancers, including leukemias, lymphomas, breast, stomach, uterine, ovarian, bladder, and lung cancers [9]. Regardless of their therapeutic value, multidrug resistance and severe cardiotoxicity are important limitations of anthracycline treatment that have prompted the discovery of novel analogues [10]. The most important anthracyclines in clinical practice are doxorubicin, daunorubicin, epirubicin and idarubicin [9]. Daunorubicin is a fermentation-derived anthracycline antibiotic that is clinically useful in the treatment of human leukemias. Daunorubicin itself is found rarely in microbial fermentations, but is present normally in the form of glycoside derivatives that yield the free drug on simple acid hydrolysis. A major by-product of daunorubicin fermentations is usually the structurally related anthracyclinone epsilon-rhodomycinone [11]. Anthracyclines including also β- and γ-rhodomycinone precursors produced by *Streptomyces spp.* [12]. These anthracyclinones were isolated since in the 1980s and could be used as precursors for the production of antibiotics with potent antimicrobial and anticancer activities [13]. Several cytotoxic metabolites were previously reported from various *S. griseus* strains including two new benzoxazines, the chandrananimycin E and dandamycin, [14] chromomycin SA and 1-(1H-indol-3-yl)-Propane-1,2,3-triol [15]. In addition, some fredericamycin A, aromatic polyketides produced by *S. griseus,* and biosynthetic genes of *S. griseus* have been heterologously cloned, expressed, and had their biological activities evaluated in vivo [16]. A recent study was conducted for the design, synthesis, and evaluation of a coherent set of configurational doxorubicin analogues, with the aim of reducing side effects and enhancing of their cytotoxic activities [17]. Therefore, this study was aimed at the production, purification, structural analysis and determination of the biological activities of two cytotoxic metabolites produced by *S. griseus* isolate KJ623766, a soil bacterial isolate previously screened in our lab that showed promising cytotoxic activities against various cancer cell lines.

## 2. Results

### 2.1. Cytotoxic Activities of the Cell Free Culture Supernatant (CFCS) Ethyl Acetate Extract

The results showed that the CFCS ethyl acetate extract has potential cytotoxic activities against both cell lines with higher activity against *Caco*2 (CD_50_ 14 µg/mL) than *Hela* cell line (CD_50_ 20 µg/mL), which indicates the presence of metabolites with higher selectivity to human colorectal adenocarcinoma (Table 1).

### 2.2. Isolation and Cytotoxic Activities of the Purified Metabolites

As determined by TLC with UV-detection and spraying with vanillin sulfuric acid spray reagent, a total of 12 major fractions (SG1-SG12) were obtained. All the fractions were subjected to 3-(4,5-dimethylthazol-2-yl)-2,5-diphenyl tetrazolium-bromide (MTT) assay to measure the cytotoxic activity of each. The fraction SG-3 (70% Hexane) showed potential cytotoxic activity against *Caco*2 and *Hela* cell lines with CD_50_ equal to 9.4 µg/mL and 12.2 µg/mL, respectively. Results of TLC indicate the presence of two major metabolites and one minor metabolite. Therefore, further purification of the active fraction SG-3 was carried out using semi-preparative HPLC, by which two pure metabolites namely, R1 and R2, were recovered and showed cytotoxic activities against *Caco*2 and *Hela* cell lines with lower activities against *Vero* cell line (Table 1).

### 2.3. Identification of the Recovered Cytotoxic Metabolites

#### 2.3.1. Metabolite R1

The metabolite R1 (5mg) was isolated as a reddish powder, ESI-MS revealed pseudomolecular ion peak at *m*/*z* 385.05 (M − H)^−^ corresponding to the molecular formula C_20_H_18_O_8_ (Figure S1). As shown in Figure S2, the 1H NMR of R1 revealed a typical pattern of anthracycline derivatives, showing three chelating hydroxyl groups at δH 12.15, 12.90, 13.61 corresponding to 4–OH, 6–OH, and 11–OH, respectively. Moreover, the spectrum revealed the presence of three adjacent aromatic protons at δH 7.93 (dd, 7.4, 1.2, 1H), δH 7.76 (m, 1H), and δH 7.37 (dd, 8.4, 1.2, 1H), corresponding to H-1, H-2, and H-3, respectively. The presence of two deshielded methine protons at δH 5.25 (m, 1H) and δH 4.91 (brs, 1H) suggested the presence of two tertiary alcohol methine protons at H-7 and H-10, respectively (Figure S2).

**Figure 1:**
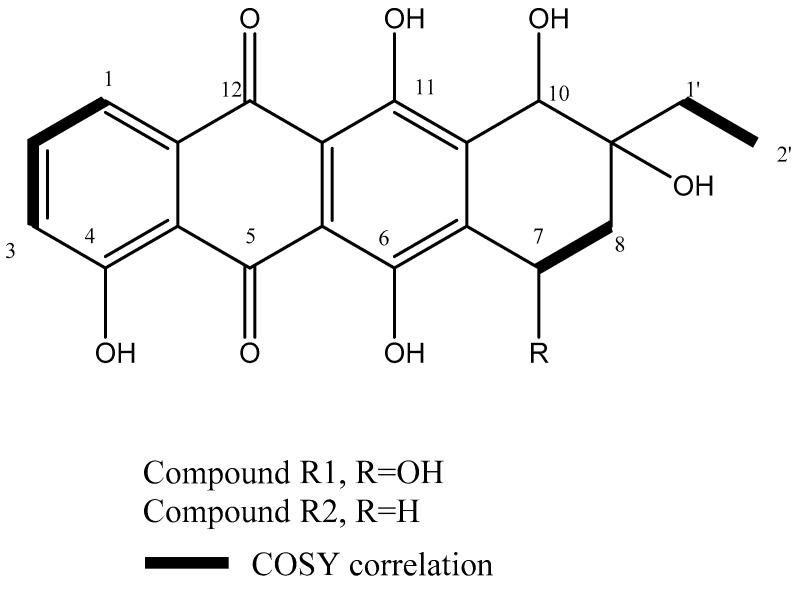
Chemical structures of the isolated compounds and COSY correlations of their corresponding protons.

This is confirmed by COSY (Figure S3) correlations where H-7 showed strong correlations with the methylene protons H-8a and H-8b at δH 2.19 (m, 1H) and δH 2.22 (m, 1H). Additionally, HSQC experiments had confirmed the assignment of protonated carbons as demonstrated in Figure S4 (Supplementary Material). The comparison of 1H NMR spectrum of R1 (Table 2) with that of known anthracyclins in the literature showed strong similarity to the known metabolite β-rhodomycinone. Therefore, the metabolite R1 was chemically identified as β-rhodomycinone (Figure 1).

#### 2.3.2. Metabolite R2

The metabolite R2 (1.5 mg), a red colored metabolite, showed an identical 1H NMR spectrum to R1 except in the replacement of H-7 at δH 5.25 (m, 1H) in R1 with the methylene protons δH 2.98 (m, 1H) and 2.90 (m, 1H), corresponding to H-7a and H-7b in R2. A comparison of 1H NMR spectrum of R2 (Table 2) with that of known anthracyclins in the literature showed strong similarity to the known metabolite γ-rhodomycinone (Figure S5). This was confirmed by COSY experiment (Figure S6) with correlations between H7 and H8. Moreover, EsI-MS had revealed a pseudomolecular ion peak at *m*/*z* 369.09 (M−H)^−^ corresponding to the molecular formula C_20_H_18_O_7_ (Figure S7). Therefore, the metabolite R2 was chemically identified as γ -rhodomycinone (Figure 1).

## 3. Discussion

Natural products are likely to provide many of the lead structures, and these will be used as templates for the construction of novel compounds with enhanced biological properties, so natural products have been the mainstay of cancer chemotherapy for the past 30 years [6]. These natural products include chemotherapeutic agents from plant, marine, and bacterial sources [7]. Although only limited numbers of the isolated compounds from microbes can be directly utilized as clinically effective medicines in their own right (naturally occurring form), streptomycetes are verified producers of anticancer drugs such as bleomycin (glycopeptides group), dactinomycin (non-ribosomal peptides group), mitomycin C (quinones group), and doxorubicin (anthracyclines group) [1]. Therefore, in this study, we aimed to isolate and identify two major metabolites of *S. griseus* isolate KJ623766, a recovered soil isolate previously screened in our lab that exhibited promising cytotoxic activity against various cancer cell lines [18]. The production was carried out in a 14L laboratory fermentor under previously determined optimum conditions [18] in order to recover sufficient pure quantities of the active metabolites as an attempt for structural elucidation. Bioassay-guided fractionation using different chromatographic techniques had led to the purification of cytotoxic metabolites coded R1 and R2 in amounts enough to identify the chemical structures of the respective metabolites using various techniques such as mass, 1D, and 2D NMR spectroscopic analysis.

For, metabolite R1, it showed potent cytotoxic activity against *Caco*2 and *Hela* cell lines, the CD_50_ were 6.3 and 9.45 µg/mL, respectively, while lower cytotoxic activity was shown against the *Vero* cell line (CD_50_ = 64.8 µg/mL). Being cancer cell lines, *Caco*2 and *Hela* cell lines have lost their normal regulation of growth or cell death and were more sensitive to the effect of cytotoxic agents than the *Vero* cell line. The lower cytotoxic effect of certain metabolites on the *Vero* cell lines usually reflects safety and potential of these metabolites to be used in future treatment of cancer. In addition, the *Vero* cell line is a continuous non-tumourigenic one when a cell passage is not prolonged [19]. Therefore, it is used to detect the cytotoxic effect of the isolated compounds and shows promising cytotoxic activity on a normal cell line. A comparison of the 1H NMR spectrum of R1 with that of known anthracyclines in the literature showed strong similarity to the known metabolite β-rhodomycinone. [20]. Therefore, the metabolite R1 was chemically identified as β-rhodomycinone.

Comparison of the 1H NMR spectrum of R2 with that of known anthracyclins in the literature showed strong similarity to the known metabolite γ-rhodomycinone [21]. Like β-rhodomycinone, γ-rhodomycinone showed potent cytotoxic activity against *Caco*2 and *Hela* cell lines, the CD_50_ were 7.11 µg/mL and 9.35 µg/mL, respectively. While lower cytotoxic activity was shown against *Vero* cell line [CD_50_ = 67.3 µg/mL). Accordingly, both β- (R1) and γ-rhodomycinone (R2) showed potent cytotoxic activities against *Caco*2 and *Hela* cell lines as compared to their activities on the *Vero* cell line, confirming their potential applicability to be used as potential anticancer agents in humans. These results matched with those of Tsuji et al. [22] which showed that γ-rhodomycinone, had no differentiation inducing activity, but was cytotoxic; however, this cytotoxic activity against normal cell lines was much lower than cancer cell lines. Potent cytotoxic activity against the *Caco*2 cell line matched with results obtained by Tan et al. [23] who examined the cytotoxic effect of ethyl acetate fraction of *Streptomyces* sp. MUM256 crude extract against Colon Cancer Cell HCT116. Results revealed that the ethyl acetate fraction of MUM256 extract was shown to exhibit the highest cytotoxicity towards HCT116 among the fractions tested, followed by the hexane fraction and the aqueous fraction as the least toxic against HCT116 cells. Lesser toxicity was observed when the extract was evaluated against a normal colon cell line CCD-18Co at all the concentrations tested in the study [23]. On the other hand, the potent cytotoxic activity against the *Hela* cell line matched with results obtained by Kim et al. [24] who evaluated the antiproliferative activities of unusaual bridged angucyclinones (Panglimycin D and Fujianmycin A), isolated from the culture of *Streptomyces* bulli GJA1, against ovarian cancer cell lines, OV90 and ES2. Results revealed potent antiproliferative activities against both cell lines [23]. Moreover, Supong and his coworkers recently showed that ε-rhodomycinone produced by a rare actinomycete Nonomuraea rhodomycinica NR4-ASC07 has a potential antimalarial activity [25].

Both β- and γ-rhodomycinone belong to the antharcycline family of drugs extracted from various *Streptomyces* spp. used in the treatment of various types of cancers [26]. They are intermediate metabolites in the biosynthetic pathways of important cytotoxic drugs such as doxorubicin, and therefore have been extracted from the various *Streptomyces* cultures that exhibited potent cytotoxic activities [27,28]. Purification of the fermentation broth from a recombinant *Streptomyces* broth yielded twelve new anthracycline antibiotics including three new ε-rhodomycinone derivatives [28]. In 2017, Kim et al. reported a combinatorial biosynthesis of glycosylated anthracyclines by co-cultivation of *Streptomyces* strains producing aglycones and nucleotide deoxysugars to yield more potent cytotoxic derivatives with high potential to be used in the treatment of various cancers in humans [27]. Anthracyclines, such as doxorubicin, are effective anticancer drugs composed of a tetracyclic polyketide aglycone and one or more deoxysugars, which play a critical role in their biological activity [28]. Moreover, *Streptomyces venezuelae*-based combinatorial biosynthetic systems have previously been developed for the production of glycosylated derivatives of doxorubicin and its biosynthetic intermediates [29]. This system was able to convert the exogenous aglycone such as ε-rhodomycinone into various glycosylated derivatives of doxorubicin or its biosynthetic intermediates [28,29].

Bacteria and bacterially produced bioactive compounds have recently emerged as a promising alternative for cancer therapeutics [30]. Bacteria like Actinobacteria are a particularly rich source of compounds with antimicrobial, anticancer, antioxidant, neuroprotective, enzymatic, and immunosuppressive functions [31]. Bacterial anticancer agents such as antibiotics, bacteriocins, non-ribosomal peptides, and polyketides showed different mechanisms of actions such as apoptosis, necrosis, reduced angiogenesis, inhibition of translation, and splicing and obstructing essential signaling pathways to kill cancer cells [30]. In this research, we were able to isolate and characterize β- and γ-rhodomycinone from the culture supernatant of an *S. griseus* isolate KJ623766. To the best of our knowledge, this the first report of β- and γ-rhodomycinone production from *S. griseus* that can be used as precursors for the construction of new derivatives with more potent cytotoxic activities. Further studies should be conducted to explore the biosynthetic pathways of the respective metabolites, as well as to produce new derivatives of the anthracycline family with higher antitumor activities and lower side effects. The latter can be achieved by structural modifications or microbial transformation of the recovered metabolites.

## 4. Materials and Methods

### 4.1. Microorganisms

Locally isolated *Streptomyces* strain from Egyptian soil sample, *S. griseus* KJ623766, was identified using 16S ribosomal RNA gene sequences (NCBI GenBank access code, KJ623766) [18]. It was deposited in the Culture Collection Ain Shams University (CCASU, Cairo, Egypt; http://www.wfcc.info/ccinfo/collection/col_by_country/e/20/, accessed on 14 September 2018) belonging to the world data center of microorganisms (WDCM) (strain number, CCASU-KJ623766). This isolate was preserved onto starch nitrate agar slants (soluble starch 10 gm, KNO_3_ 2 gm, K_2_HPO_4_ 1 gm, NaCl 0.5 gm, MgSO_4_.7H_2_O 0.5 gm, *CaCO*_3_ 3 gm, agar 20 gm, FeSO_4_.7H_2_O 0.1 gm, MnCl_2_ 4H_2_O 0.1 gm, ZnSO_4_ 7H_2_O 0.1 gm per 1 L of distilled water) and sub-cultured every month, while slant-medium (50:50) (tryptone 10 g, yeast extract 5 g, glycerin 500 mL and distilled water up to 1 L) was used for long term preservation.

### 4.2. Cell Lines

Three cell lines, including kidney and epithelial cells derived from the African green monkey (*Vero* cell line, ATCC No.CCL-81), colorectal adenocarcinoma derived from the human colon (*Caco*2 cell line), and epithelioid carcinoma derived from the human cervix (*Hela* cell line) were obtained from VACSERA, Egypt. The *Caco*2 and *Hela* cell lines were used to study the cytotoxic activities of the crude extract and isolated metabolites; stock cultures of these cell lines were grown in T-75 tissue culture flasks containing 20 mL of RPMI-1640 medium with 1% antibiotic antimycotic solution and 10% fetal bovine serum. The medium was changed at 48 h intervals and cells dissociated with trypsin solution (0.25% in phosphate buffer saline). *Vero* cell line is a continuous non-tumourigenic when a cell passage was not prolonged and was used to analyze the cytotoxic activities of isolated metabolites [12]. It was propagated in Eagle minimum essential medium (EMEM) with Hank’s balanced salt solution (HBSS), supplemented with 10% Fetal bovine serum (FBS) and antibiotics (penicillin 100 IU and streptomycin/mL 100 IU) solution, and maintained in EMEM with Earl’s balanced salt solution (EBSS) supplemented with 2% FBS and antibiotics solution [19].

### 4.3. Production and Isolation of Cytotoxic Agent(s) Produced by S. griseus KJ623766

#### 4.3.1. Preparation of Seed Culture

This was carried out according to Peng et al. [32] with some modifications as follows; surface inoculation of the tested isolate onto starch nitrate agar slant was carried out. After 7 days of incubation at 28 °C, the formed spores were scalped from the agar surface and suspended in 3 mL distilled water. An aliquot (200 µL) of the resulting spore bacterial suspension was used to inoculate 10 mL of the soybean meal medium (soybean 15 g, glucose 15 g, NaCl 5 g, and CaCO_3_ 1 g per 1 L of distilled water in 100 mL flask) and incubated at 28 °C with shaking 200 RPM for 72 h in a C5KC shaking incubator (New Brunswick scientific, Edison, NJ, USA). The culture obtained was used to inoculate the main culture.

#### 4.3.2. Extraction of Cytotoxic Metabolite(s)

1 L of CFCS was extracted with ethyl acetate at the level of 1:1 (*v*/*v*) in subsequent manner [33,34,35], and the collected organic layers were evaporated using rotary evaporator (Heidolph instruments GmbH and Co. Schwabach, Germany) under vacuum at 45 °C to yield 600 mg of the reddish brown residue. After complete evaporation, representative sample of the fraction residue was re-dissolved in5 % DMSO in tissue culture medium. The cytotoxic activity of the re-dissolved fraction was evaluated against *Caco*2 and *Hela* cancer cell lines using MTT assay [36]. As previously reported and according to the screening program of American National Cancer Institute (NCI), a crude extract is generally considered to have in vitro cytotoxic activity if the CD_50_ value is ≤30 μg/mL [37].

#### 4.3.3. Recovery of Cytotoxic Metabolite(s)

**Figure 2:**
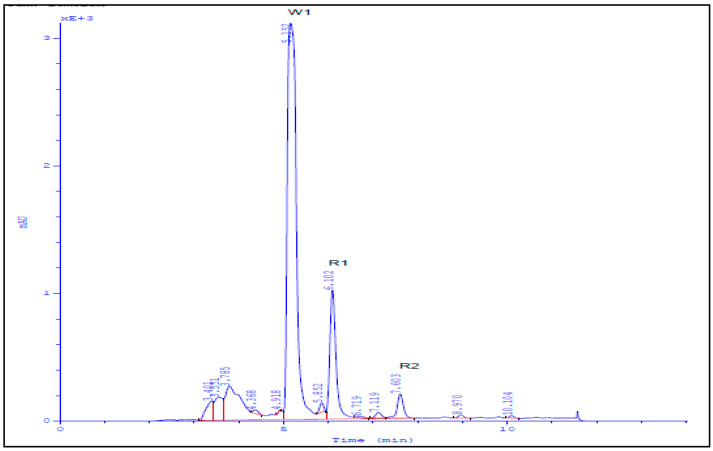
HPLC chromatogram of isolated compounds R1 and R2.

Medium pressure chromatographic separations were carried out with a PuriFlash 4100 system (Interchim; Montluçon, France) consisting of a mixing HPLC quaternary pump, a PDA-UV-Vis detector 190–840 nm, a fraction collector, and a sample loading module. For system controlling and process monitoring, Interchim Software 5.0 was used. 360 mg sample was dissolved in 50 mL of methanol then introduced into the column via dry load using 4 gm silica. Elution started with hexane:ethyl acetate (100%:0%) followed by ethyl acetate: methanol (100%:0%) gradient to yield 117 fractions. Elution was monitored using TLC (normal phase silica gel precoated plates F254, Merck, Germany) with UV-detection and spraying with vanillin sulphuric acid spray reagent to yield 12 major fractions (SG1-SG12); these fractions were subjected to MTT assay to measure the cytotoxic activity of each against *Caco*2 and *Hela* cell lines. Final purification steps were performed using preparative HPLC (Knauer, Germany) on Kromasil ODS preparative column (10 mm × 250 mm) at flow rates of 4 mL/min and UV detection at 254, yielding metabolites R1 and R2 (Figure 2). MTT assay was carried out to measure the cytotoxic activity of each against *Vero*, *Caco*-2, and *Hela* cell lines [36].

#### 4.3.4. Fermentation in a Laboratory Fermenter

This was done according to Radwan et al. [33] with some modifications, where the fermentation process was conducted in a 14 L CelliGen 310 bioreactor (New Brunswick Scientific, Edison, NJ, USA), with its Reactor Process Control (RPC) software. The fermentation process was carried out using a 5 L working volume of soybean meal medium under a condition of 28 °C incubation temperature; 200 RPM agitation speed, 3 volume/volume/minute (vvm), i.e., 15 standard liter per minute (SLPM) aeration rate and 2 bar airflow pressure. The dissolved oxygen concentration was adjusted to obtain 100% saturation at the beginning of the run and dissolved oxygen (DO) percentage was sensed by the DO probe and monitored during the fermentation process. The pH was adjusted to 7 at the beginning of the run, and remained uncontrolled during the fermentation process (72 h). After inoculation with 3-day-old culture of the tested isolate (5% *v*/*v*) under aseptic conditions, the run started under the previously stated conditions. The fermentation process was left for 72 h, during which foam was suppressed using silicon oil. The culture obtained was centrifuged at 6000 RPM for 10 min using an EBA20 Centrifuge (Hettrich GmbH, Taufkirchen, Germany), and the cell free culture supernatant (CFCS) obtained was collected.

### 4.4. Spectroscopic Analysis of the Recovered Cytotoxic Metabolites

Structures of the isolated metabolites were determined based on LC-ESI-MS analysis performed using waters^®^xevo-tqd^®^ (UPLC/MS/MS (p/n 186005453, IET, Mundelein, IL, USA) and NMR spectroscopic analysis, which was recorded on a Bruker AVANCE HD III 400 MHz spectrometer (Bruker AG, Faellanden, Switzerland). NMR samples were dissolved in methanol-d4 or chloroform-d3 (Sigma Aldrich, Germany) and transferred to 3 mm NMR tubes (Bruker AG, Faellanden, Switzerland).

### 4.5. Evaluation of the Cytotoxic Activities of the Recovered metabolites

An MTT assay was carried out as previously reported [20] with some modifications; 100 μL of the tested metabolite (1 mg of the metabolite dissolved in 5% DMSO and tissue culture medium) was added to a well that contained 100 μL of tissue culture medium, followed by a two-fold serial dilution. A total of 12 dilutions were used for each metabolite to calculate CD_50_ for each. Control wells contained two aliquots of 100 μL of ethyl acetate extract of soybean meal medium (1 mg dissolved in 5% DMSO and tissue culture medium) and 100 μL of tissue culture medium, followed by two-fold serial dilutions. Values were compared to those of the positive control doxorubicin. After 24 h incubation period at 37 °C in a CO_2_ incubator, the wells were washed with PBS, followed by incubation with 100 μL MTT solution (1 mg/mL) per well for 1 h at 37 °C. Supernatants were obtained by decantation and the cells were mixed with 100 μL DMSO per well to dissolve formazan particles. Elutes of the 12 wells of each tested metabolite were obtained and measured spectroscopically at 540 nm using differential wavelengths of 630 nm in a Plato R496 Microplate reader AMD diagnostics, Graz, Austria. Control wells were similarly handled, and the percentage cytotoxicity was calculated by the following formula [36].

Cytotoxicity% = 1 − {A540 of test culture/A540 of control culture} × 100

## 5. Conclusions

This study targeted large scale production of cytotoxic metabolites by *S. griseus* isolate KJ623766 in a 14L fermenter and studied the different recovery methods for downstream processing. The structures of isolated metabolites were determined based on the mass, 1D, 2D NMR, COSY, and HSQC BMR spectroscopic analysis and identified as β- and γ-rhodomycinone, respectively. The respective metabolites were biologically analyzed using various cancer cell lines for their cytotoxic actives and exhibiting promising cytotoxic activities. Accordingly, *S. griseus* isolate KJ623766 could be used as a potential industrial strain for the commercial production of β- and γ-rhodomycinone to be used for production of different derivatives of antitumor antibiotics of the anthracycline family.

## Figures and Tables

**Table 1:** Cytotoxic activities of ethyl acetate extract and isolated metabolites against different cell lines.

Tested Metabolite	Average Cytotoxic Activity (CD_50_) against Different Cell Lines (µg/mL) ±SD		
	*Caco*2	*Hela*	*Vero*
Ethyl acetate extract	14 ± 0.88	20 ± 0.52	nd
Fraction SG-3	9.4 ± 0.63	12.2 ± 0.62	nd
R1	6.3 ± 0.35	9.45 ± 0.25	64.8 ± 0.88
R2	6.3 ± 0.35	9.35 ± 0.45	67.3 ± 0.37
doxorubicin	1.25 ± 0.5	1.7 ± 0.6	nd

nd, not determined.

**Table 2:** NMR data of metabolites R1, R2, and standard γ-rhodomycinone in the literature. Chemical shifts are expressed in δH values (ppm) from internal TMS. Coupling constants in parentheses are given in *J* in Hz.

Position	δ_H_ (CDCl_3_, 400 MHz, *J* in Hz), Metabolite R1	δ_H_ (CDCl_3_, 400 MHz, *J* in Hz), Metabolite R2	δ_H_ (CDCl_3_)γ-Rhodomycinone (Standard)
1	7.93 (dd, 7.4, 1.2, 1H)	7.91 (dd, 7.8, 1.0, 1H)	7.89 (d, 6.6, 1.1, 1H)
2	7.76 (t, 8.0, 1H)	7.73 (t, 8.0, 1H)	7.72 (t, 8.07, 1H)
3	7.37 (dd, 8.4, 1.2, 1H)	7.35 (dd, 8.2, 1.0, 1H)	7.31 (d, 7.33, 1.1, 1H)
7	5.26 (m, 1H)	7a: 2.98 (m, 1H), 7b: 2.90 (m, 1H)	5.24 (d, 5.14)
8a	2.19 (m, 1H)	1.98 (m, 1H)	2.29–2.14 (m, 2H)
8b	2.22 (m, 1H)	2.35 (m, 1H)	
10	4.91 (s, 1H)	4.82 (s, 1H)	5 (s, 1H)
4-OH	12.15 (s, 1H)	12.27 (s, 1H)	12.20 (s, 1H)
6-OH	12.91 (s, 1H)	12.77 (s, 1H)	12.90 (s, 1H)
11-OH	13.61 (s, 1H)	13.87 (s, 1H)	13.60 (s, 1H)
1′a	1.81 (m, 1H)	1.95 (m, 1H)	1.74–1.85 (m, 2H)
1′b	1.92 (m, 1H)	2.00 (m, 1H)	
2′	1.15 (t, 7.5, 3H)	1.13 (t, 7.5, 3H)	1.12 (t, 7.34, 3H)

## Data Availability

The data presented in this study are available in the main manuscript as well as in the Supplementary Materials.

## Supplementary Materials

The following are available online: Figure S1—ESI-MS of isolated compound R1; Figure S2—Proton NMR spectrum of R1; Figure S3—COSY NMR spectrum of R1; Figure S4—HSQC NMR spectrum of R1; Figure S5—Proton NMR spectrum of R2; Figure S6—COSY NMR spectrum of R2; Figure S7—ESI-MS spectrum of compound R2.

## Sample Availability

Samples of the β- and γ-rhodomycinone compounds are available from the authors.
