# Supplementary material for: Streptomyces griseus KJ623766: A Natural Producer of Two Anthracycline Cytotoxic Metabolites β- and γ-Rhodomycinone
Source: Molecules. 2021 Jun 30;26(13):4009. doi: 10.3390/molecules26134009 (PMC8271628; doi:10.3390/molecules26134009)
Supplement: Supplementary file 1 [file molecules-26-04009-s001.zip › molecules-1247638-supplementary.pdf]

Supplementary Materials

Streptomyces Griseus KJ623766: A Natural Producer of Two Anthracycline Cytotoxic Metabolites β- and γ-rhodomycinone

Ahmed S. Abu Zaid [1], Ahmed E. Aleissawy [2], Ibrahim S. Yahia [3,4,5], Mahmoud A. Yassien [1], Nadia A. Hassouna [1] and Khaled M. Aboshanab [1,*]

[1] Department of Microbiology and Immunology, Faculty of Pharmacy, Ain Shams University, Organization of African unity st, P.O. Box 11566, Abbassia, Cairo, Egypt; ahmed.abouzid@pharma.asu.edu.eg (A.S.A.Z.); mahmoud.yassien@pharma.asu.edu.eg (M.A.Y.); nadia.hassouna@pharma.asu.edu.eg (N.A.H.)

[2] Department of Pharmacognosy, Faculty of Pharmacy, Ain Shams University, Organization of African unity st, P.O. Box 11566, Abbassia, Cairo, Egypt; aelissawy@pharma.asu.edu.eg

[3] Research Center for Advanced Materials Science (RCAMS), King Khalid University, Abha 61413, P.O. Box 9004, Saudi Arabia; ihussein@kku.edu.sa

[4] Advanced Functional Materials & Optoelectronic Laboratory (AFMOL), Department of Physics, Faculty of Science, King Khalid University, P.O. Box 9004, Abha, Saudi Arabia

[5] Nanoscience Laboratory for Environmental and Bio-Medical Applications (NLEBA), Semiconductor Lab., Physics Department, Faculty of Education, Ain Shams University, Roxy, 11757 Cairo, Egypt

* Correspondence: aboshanab2012@pharma.asu.edu.eg; Tel.: (202) 010-758-2620

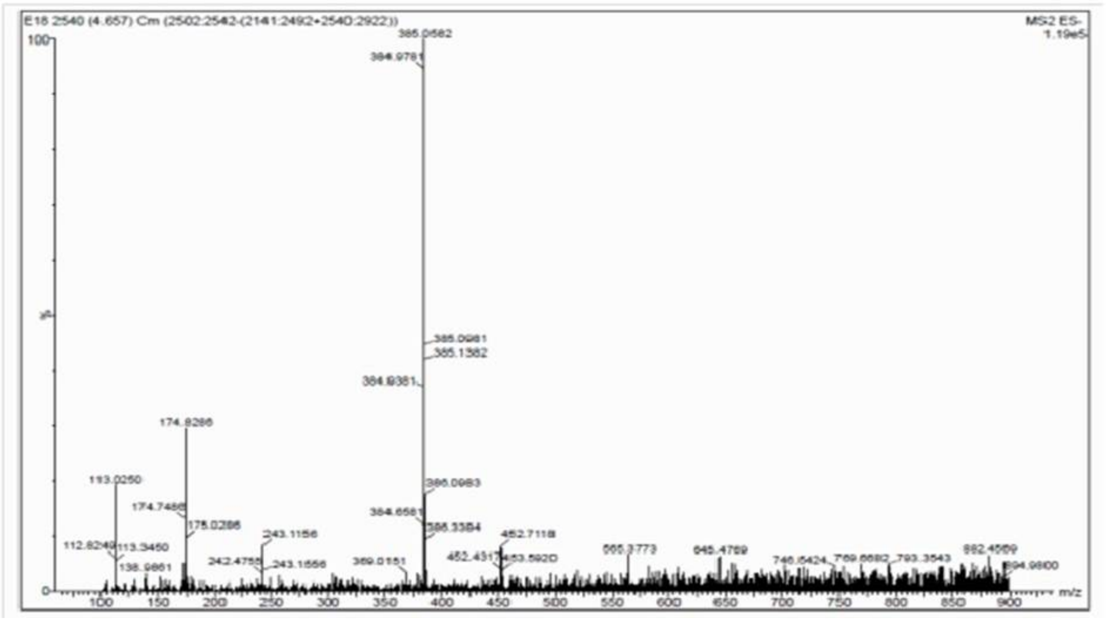

Figure S1. ESI-MS spectrum of compound R1

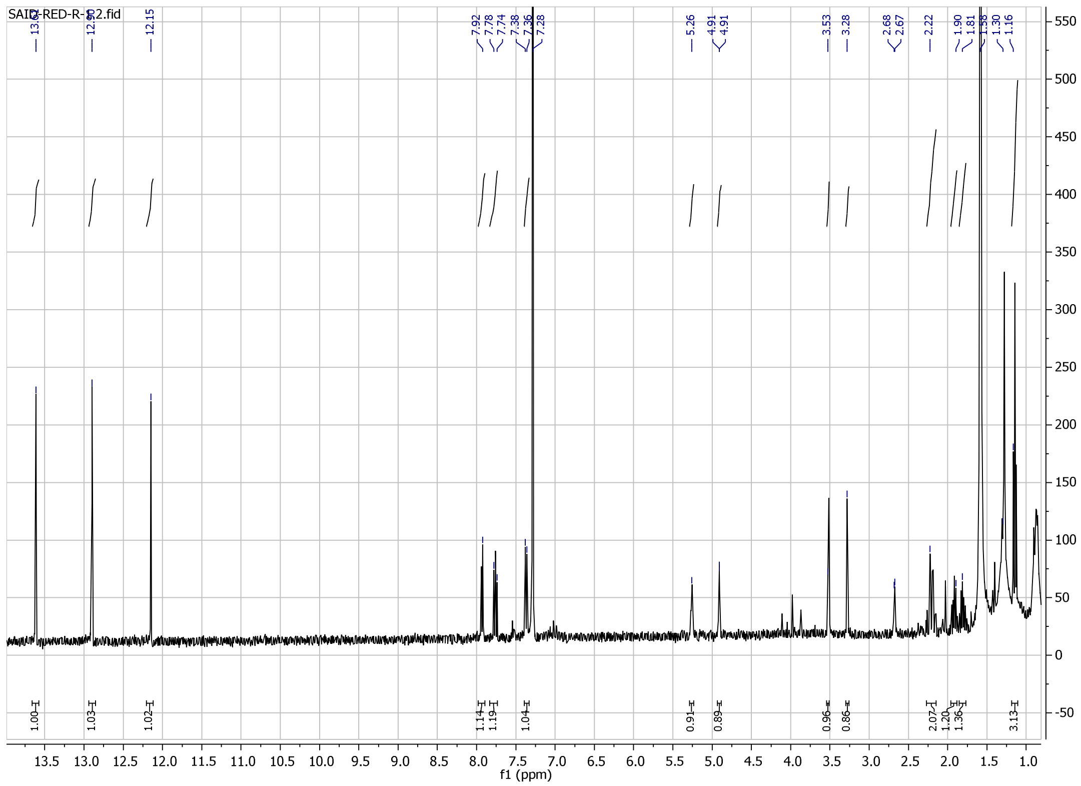

Figure S2. Proton NMR spectrum of R1

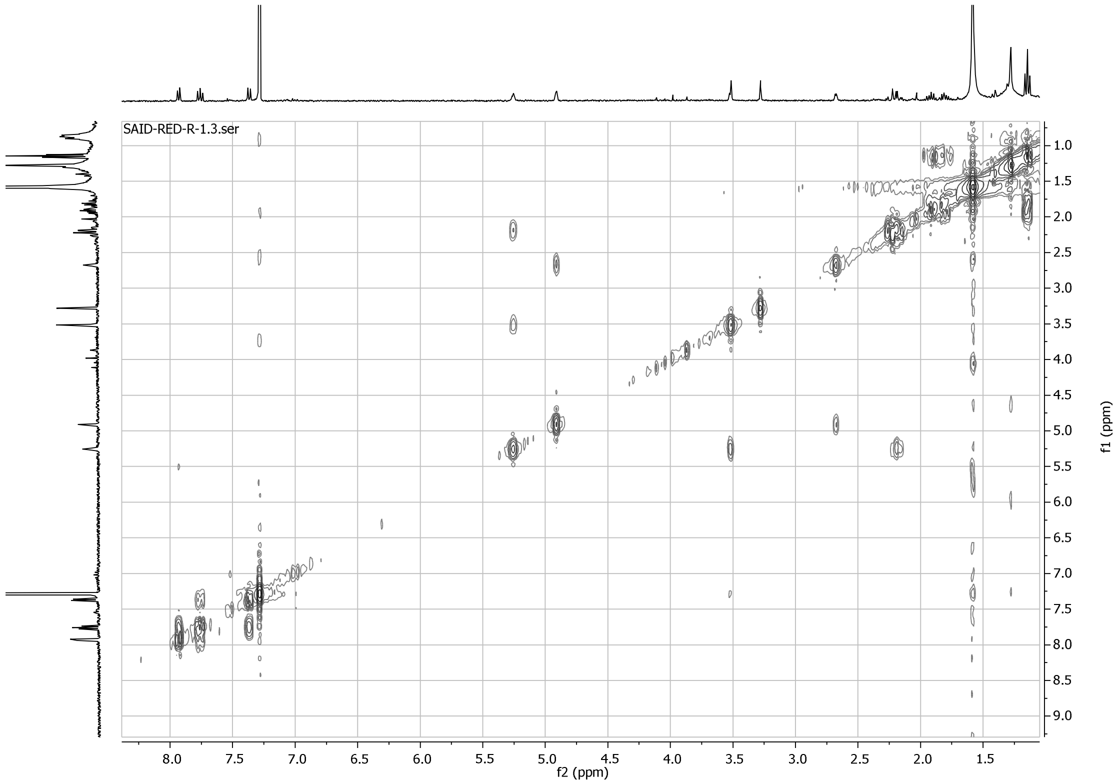

Figure S3. COSY NMR spectrum of R1

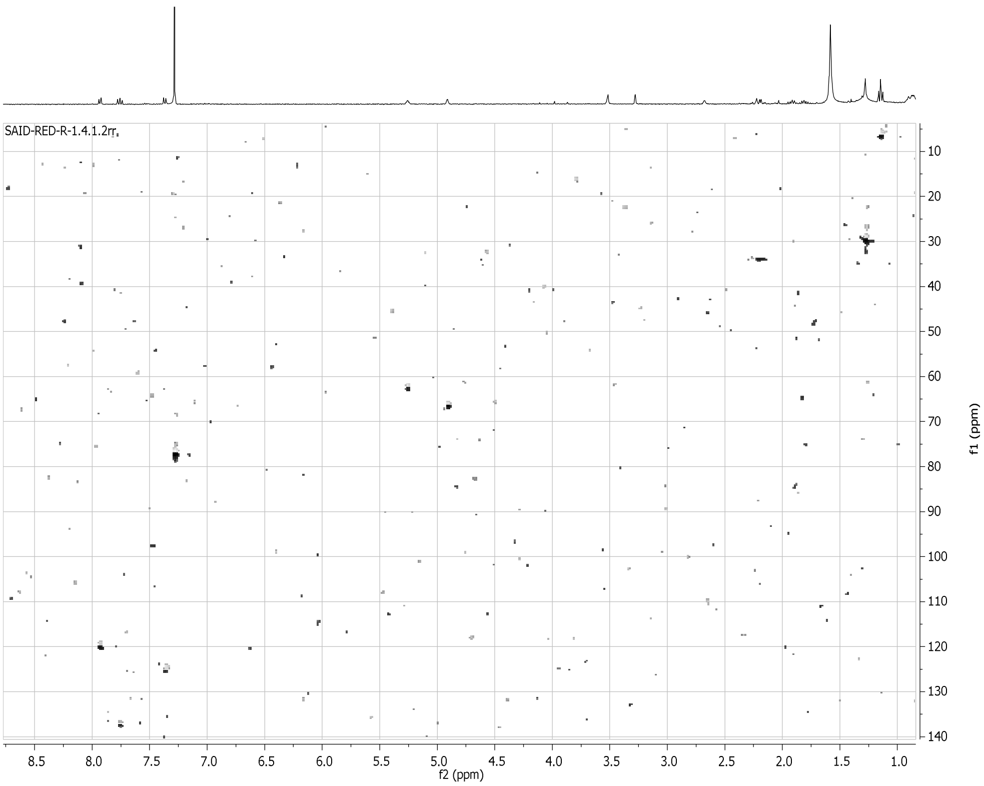

Figure S4. HSQC NMR spectrum of R1

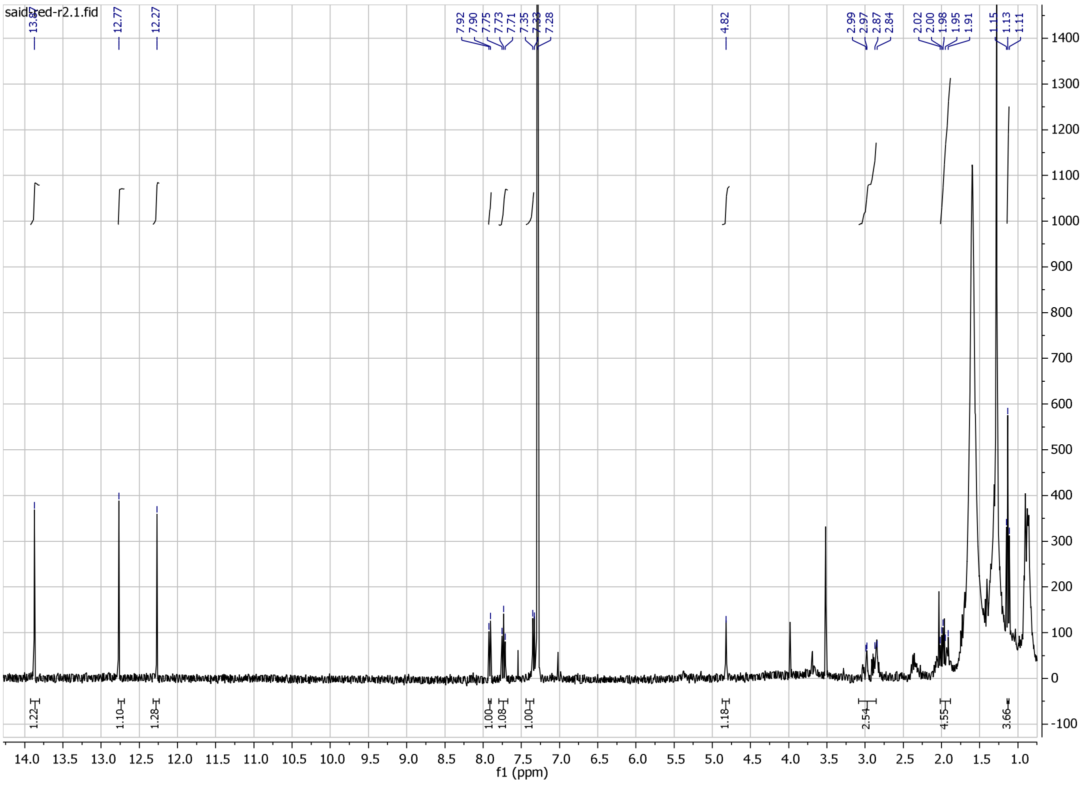

Figure S5. Proton NMR spectrum of R2

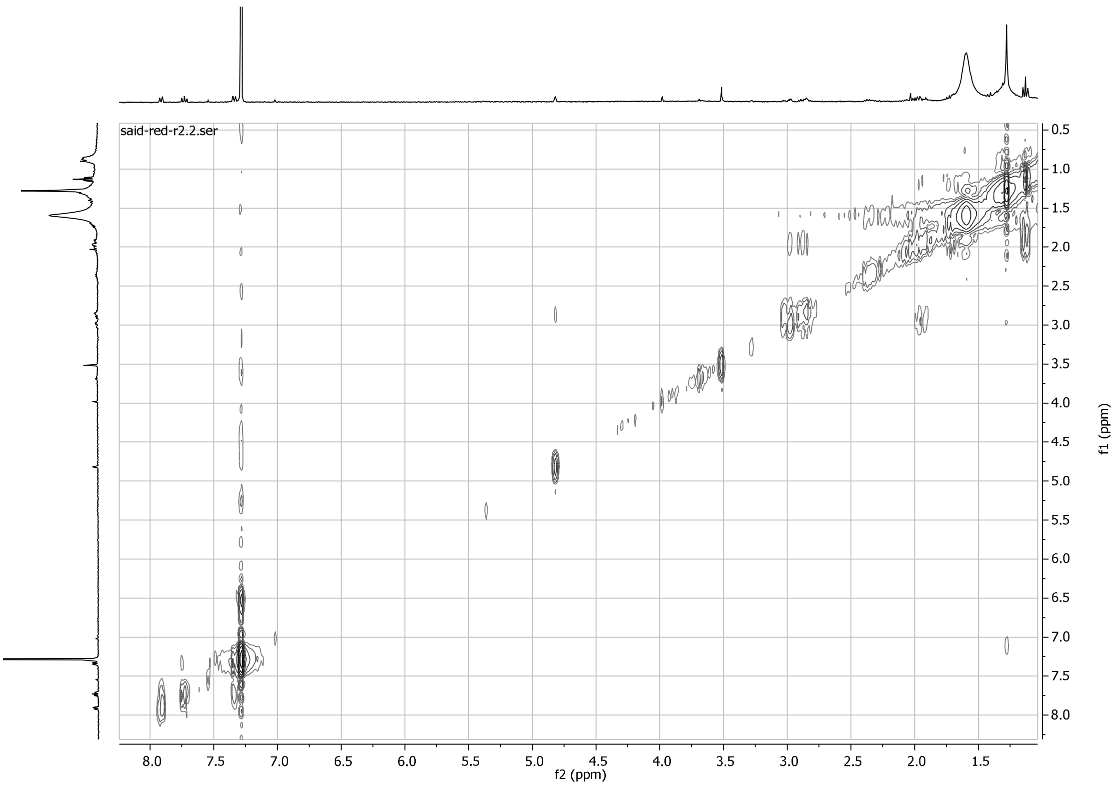

Figure S6. COSY NMR spectrum of R2

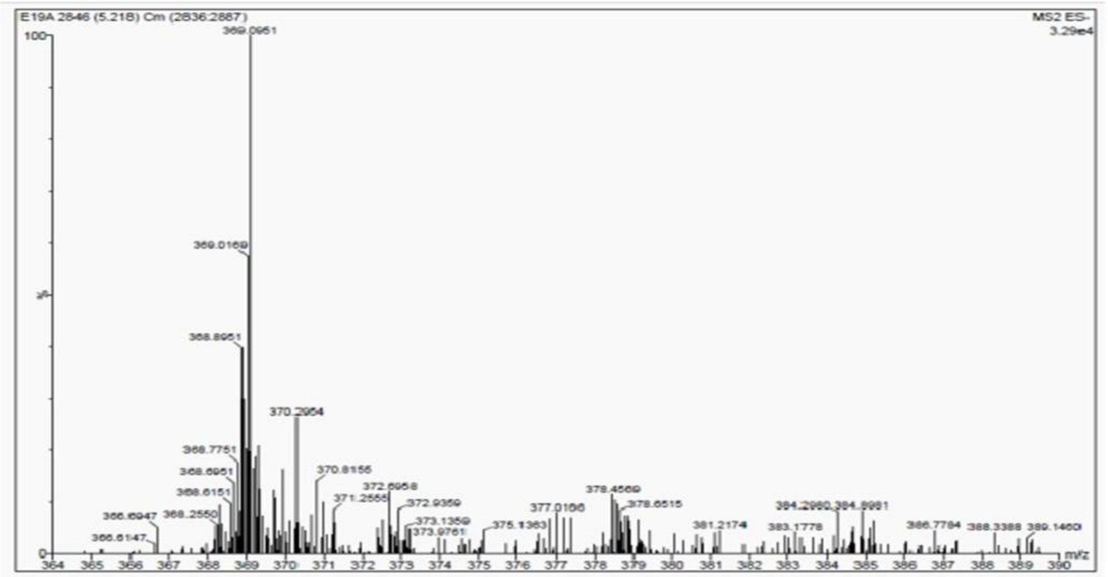

Figure S7. ESI-MS spectrum of compound R2
